# The HLA library for drug screening in preventing severe drug hypersensitivity

**DOI:** 10.1186/2045-7022-4-S3-P51

**Published:** 2014-07-18

**Authors:** Chun-yu Wei

**Affiliations:** 1 Institute of Biomedical Sciences/Academia Sinica, Taiwan

## Background

The clinical and economic impact of severe drug hypersensitivity is huge for both healthcare and pharmaceutical industry; however, no method for predicting culprit candidate t drugs under clinical development exists currently. As genetic variants in human-leukocyte antigens (HLA) have been linked to inter-individual differences in the risk of drug hypersensitivity, we ascertained whether HLA library can serve as a drug screening tool for their potential to cause severe drug hypersensitivity.

## Method

We cloned and generated stable cell lines expressing one common HLA allele, and then purified each HLA protein genetically linked to particular hypersensitivity, immobilized on a protein chip and analyzed the interaction between HLA and drugs by surface plasmon resonance (SPR) analysis.

## Results

With HLA library coating on the chip, the direct interaction between specific HLA protein complexes and culprit drugs were detected by SPR analysis. For example, HLA-B*1502 protein interacted with aromatic anti-epileptic drugs, such as carbamazepine (CBZ), CBZ analogs (10,11-eposide CBZ, oxcarbazepine, licabazepine and eslicarbazepine), phenytoin (PHT), and lamotrigine (LTG), but not structure-unrelated compounds, such as gabapentin (GBP), levetiracetam (LEV), and topiramate (TPN); In addition to HLA-B*1502, other HLA-B75 members could also present CBZ, whereas HLA-B62 and HLA-B72 members could not, consistent with pharmacogenetic data. Moreover, HLA-B*5801 binds to allopurinol and oxypurinol, but not febuxostat. Similar, HLA-B*5701 could interact with the nucleoside reverse transcriptase inhibitor, abacavir, but not the non-nucleoside reverse transcriptase inhibitor, nevirapine.

## Conclusion

These data suggested the possibility of using HLA library which contains different HLA molecules for its ability to bind drug as a screen tool to screen drugs for their potential to cause severe drug hypersensitivity.

